# Molecular signature comprising 11 platelet-genes enables accurate blood-based diagnosis of NSCLC

**DOI:** 10.1186/s12864-020-07147-z

**Published:** 2020-10-27

**Authors:** Chitrita Goswami, Smriti Chawla, Deepshi Thakral, Himanshu Pant, Pramod Verma, Prabhat Singh Malik, Jayadeva ▮, Ritu Gupta, Gaurav Ahuja, Debarka Sengupta

**Affiliations:** 1 Department of Computer Science and Engineering, Indraprastha Institute of Information Technology, New Delhi, India; 2 Department of Computational Biology, Indraprastha Institute of Information Technology, New Delhi, India; 3 Laboratory Oncology Unit, All India Institute of Medical Sciences, New Delhi, India; 4 Department of Electrical Engineering, Indian Institute of Technology, New Delhi, India; 5 Department of Medical Oncology, All India Institute of Medical Sciences, New Delhi, India; 6 Institute of Health and Biomedical Innovation, Queensland University of Technology, Brisbane, Australia; 7 Centre for Artificial Intelligence, Indraprastha Institute of Information Technology, New Delhi, India

**Keywords:** Liquid biopsy, Tumour educated platelet, NSCLC, Molecular diagnostics, Gene-signature

## Abstract

**Background:**

Early diagnosis is crucial for effective medical management of cancer patients. Tissue biopsy has been widely used for cancer diagnosis, but its invasive nature limits its application, especially when repeated biopsies are needed. Over the past few years, genomic explorations have led to the discovery of various blood-based biomarkers. Tumor Educated Platelets (TEPs) have, of late, generated considerable interest due to their ability to infer tumor existence and subtype accurately. So far, a majority of the studies involving TEPs have offered marker-panels consisting of several hundreds of genes. Profiling large numbers of genes incur a significant cost, impeding its diagnostic adoption. As such, it is important to construct minimalistic molecular signatures comprising a small number of genes.

**Results:**

To address the aforesaid challenges, we analyzed publicly available TEP expression profiles and identified a panel of 11 platelet-genes that reliably discriminates between cancer and healthy samples. To validate its efficacy, we chose non-small cell lung cancer (NSCLC), the most prevalent type of lung malignancy. When applied to platelet-gene expression data from a published study, our machine learning model could accurately discriminate between non-metastatic NSCLC cases and healthy samples. We further experimentally validated the panel on an in-house cohort of metastatic NSCLC patients and healthy controls via real-time quantitative Polymerase Chain Reaction (RT-qPCR) (AUC = 0.97). Model performance was boosted significantly after artificial data-augmentation using the EigenSample method (AUC = 0.99). Lastly, we demonstrated the cancer-specificity of the proposed gene-panel by benchmarking it on platelet transcriptomes from patients with Myocardial Infarction (MI).

**Conclusion:**

We demonstrated an end-to-end bioinformatic plus experimental workflow for identifying a minimal set of TEP associated marker-genes that are predictive of the existence of cancers. We also discussed a strategy for boosting the predictive model performance by artificial augmentation of gene expression data.

## Background

Invasive, solid tissue-based confirmatory diagnosis of cancer suffers from several shortcomings, including surgical tissue acquisition, provision for resampling, and the risk of infection/bleeding [1, 2]. Further, it just offers a one-time snapshot of the disease life-cycle, obscuring the leads for potential course-corrections. Liquid biopsy methods have emerged as promising alternatives, aimed at overcoming these limitations [3–5]. Tumor-derived blood-based biomarkers often hold valuable information about their malignant origins. Some of the commonly used cancer biomarkers isolated from peripheral blood include cell-free DNA (cf-DNA) [6, 7], circulating endothelial cells (CEC) [8, 9] and circulating tumor cells (CTC) [10]. These methods, however, suffer from high type 2 error rates. Despite many promises, none of these blood-based bio-sources could so far be effectively used for early cancer detection. Different cancers have shown varying degrees of false-positive and false-negative rates when using CTC and ctDNA based detection [11].

NSCLC, the most prevalent form of lung cancer, is largely asymptomatic in its early stage. The majority of its detection takes place at an advanced stage when the disease has spread widely to distant organs. As such, the development of affordable early diagnostic tests plays a major role in improved management of the disease. For NSCLC, some studies have shown up to 100% false positives CTC detection rates in patient samples [12]. Jenkins and colleagues reported false-negative rates upto 50% in patients with intra-thoracic limited (M1a) disease while using a ctDNA-based method [13]. A recent study by Best et al. [4], revealed significant changes in platelet transcriptomes between cancer patients and healthy individuals, which led to the new concept of Tumor Educated Platelets (TEPs). The dramatic changes in platelet transcriptome have, since, been linked to the cross-talk between tumor cells and platelets [14]. Using ∼1000 variable genes, the authors reported 96% accuracy in distinguishing localized and metastatic tumors of six major cancer types from healthy cases [4]. A study by Best and colleagues [4] showed that TEPs are substantially more accurate in predicting the existence of cancer with false-negative and false-positive rates recorded as 4% and 8% respectively. In an independent study focusing on Non-Small Cell Lung Cancer (NSCLC), the authors designed a classification model derived from ∼1600 genes and reported an overall accuracy of 88% for late-stage cancer and 81% for locally advanced cancer by employing statistical and machine learning-based techniques [15]. More recently, Sheng and colleagues leveraged the RNA sequencing (RNA-seq) dataset published by Best et al. [15] to achieve 88.9% accuracy for NSCLC classification with a mere 48 genes. Their work highlighted the scope of retaining predictability with a concise gene-panel, thereby inspiring its potential diagnostic use [16]. While such informative explorations extend the field, due to lack of validation, they seldom see materialization.

To address the above issues and to fully exploit the potential of TEPs for accurate and economical detection of cancer, we developed a practical computational cum cross-assay experimental validation workflow which accounts for small sample sizes. As part of this study, we used a publicly available RNA-Seq dataset and extracted 11 informative genes that help distinguish between cancer and healthy samples. The performance of the gene-set was tested on an independent RNA-Seq data comprising 57 early locally advanced NSCLC patients (non-metastatic) and 377 healthy individuals [15]. Our gene panel perfectly distinguished between the two classes (AUC = 1). We also experimentally validated the effectiveness of these genes on a geographically distinct cohort of NSCLC patients (10 NSCLC patients, 7 healthy donors) using RT-qPCR. In many clinical settings, the turn around time of sample acquisition is high. This hinders experimental validation in case of proof of concept studies. To overcome this limitation, we augmented the training data with artificial patient and healthy samples, which led to near-perfect identification of the NSCLC cases (AUC = 0.99).

## Results

### A set of 11 platelet genes reliably discriminates cancers and healthy controls

**Fig. 1:**
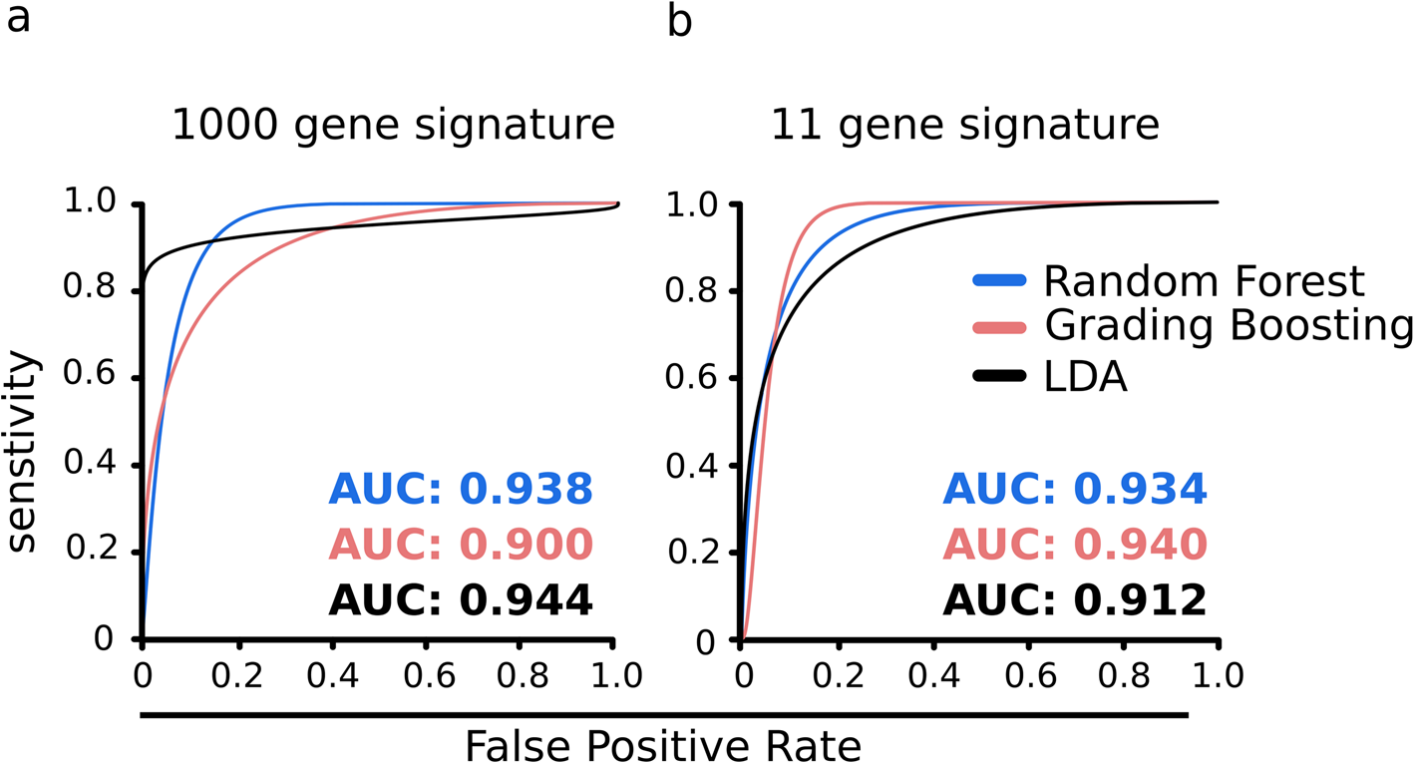
Panel of 11 genes performs equivalent to panel of 1000 genes. AUC (Area under the curve) plots representing the comparative performance of 1000 gene and 11 gene panels respectively on platelet transcriptomes from healthy and NSCLC patients. The predictive power of the gene-sets was evaluated using three widely used classification algorithms namely Gradient Boosting Machines (GB), Random Forest (RF), and Linear Discriminant Analysis (LDA)

Tumor Educated Platelets opened a new frontier in liquid biopsy research [4]. Since the introduction, several studies have been published developing multivariate classification models for molecular stratification of cancers and healthy controls [3, 15, 17]. Most of these studies made use of several hundreds of genes to attain decent accuracy levels. Profiling large numbers of genes incur a significant cost, impeding its diagnostic adoption. We asked if the gene-set can be narrowed down, without compromising on the disease predictability. We analyzed a published, multi-cancer RNA-Seq data [4], and came up with a set of 11 platelet genes (*CD79B, CSDE1, IL-32, ITGA2B, LUC7L, NDUFAB1, RBM6, SKAP2, SS18L2, TRAF3IP3, and ZNF195)* that enables accurate classification of cancer and healthy samples (refer Methods). We used Gradient Boosting Machines (GB), Random Forest (RF) and Linear Discriminant Analysis (LDA), three widely used classification methods to assess the potential of these genes in classifying cancer and healthy blood specimens. The best cross-validation accuracy was obtained using the GB classifier (AUC = 0.94), which matched the performance of the models that used 1000 variables, going by the recommendations of Best and colleagues ([4], Fig. 1, Table S1). Notably, the selection of these 11 genes was not biased to any particular cancer, and, therefore, can be used across at least four other cancer types other than non-small-cell lung cancer (NSCLC). These include colorectal cancer (CRC), glioblastoma multiforme (GBM), breast cancer (BRCA), and pancreatic cancer (PC). In the case of hepatobiliary cancer (HBC), our accuracy estimates are not reliable due to the lack of samples (*n*=5). It should also be noted that for cancer types other than NSCLC, the gene panel was not validated on independent cohorts of patient samples. Therefore the performance metrics, though promising, may not be considered conclusive for cancers types besides NSCLC.

### Validation of the gene panel in lung cancer patients

**Fig. 2:**
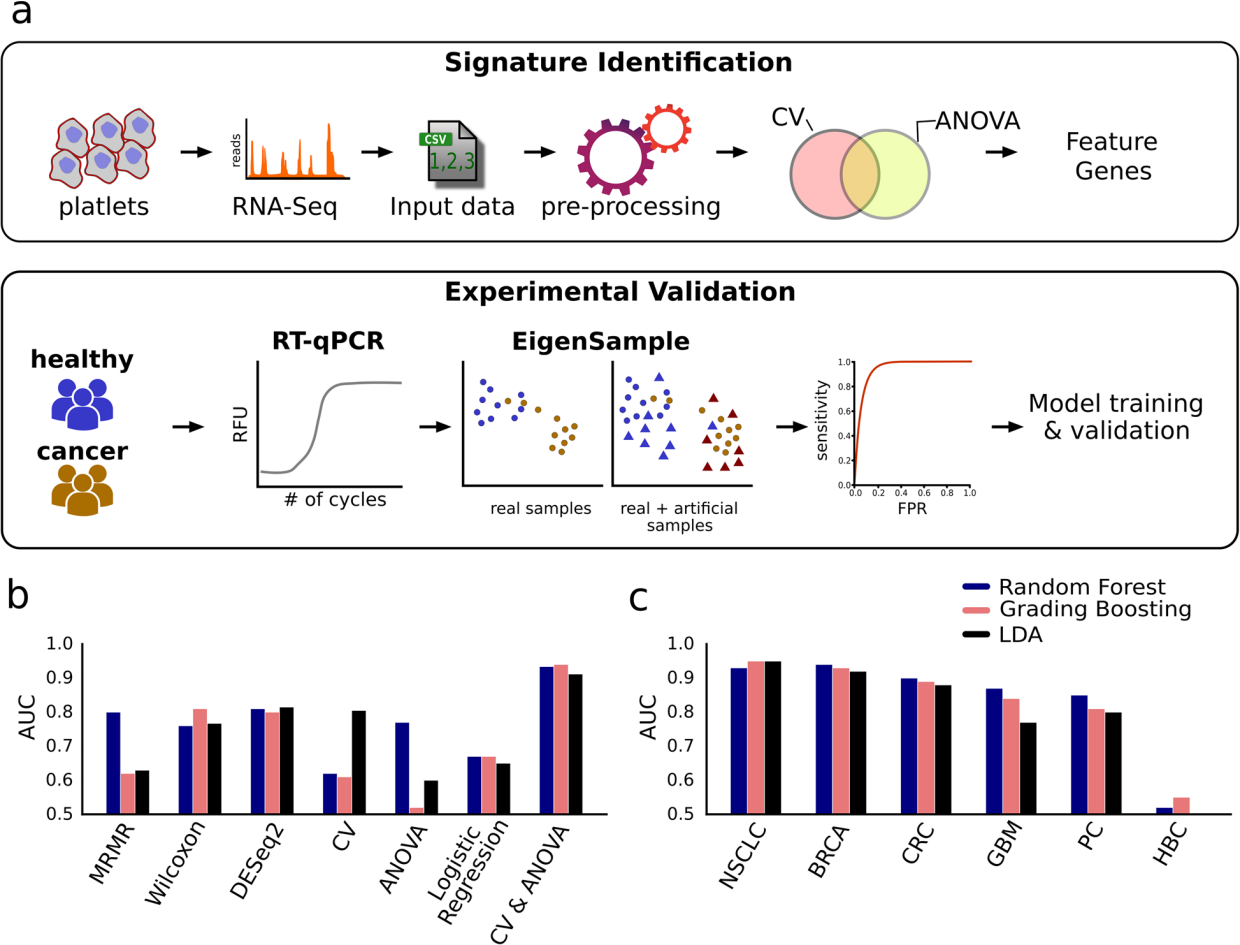
Schematic representation of workflow and discovery of gene-signature. (**a**) The upper panel is a schematic representation illustrating the underlying methodology implemented for the identification of the concise gene-panel utilizing RNA-seq data of Tumor Educated Platelets (TEPs) (GSE68086). The lower panel represents the experimental design and the downstream statistical analysis employed in the validation of the inferred signature on a geographically distinct NSCLC patient cohort. (**b**) A comparison between different feature selection methods shows that a combination of Coefficient of Variation (CV) and Analysis of Variance (ANOVA) performs the best. (**c**) Classification accuracy across different cancer types

**Fig. 3:**
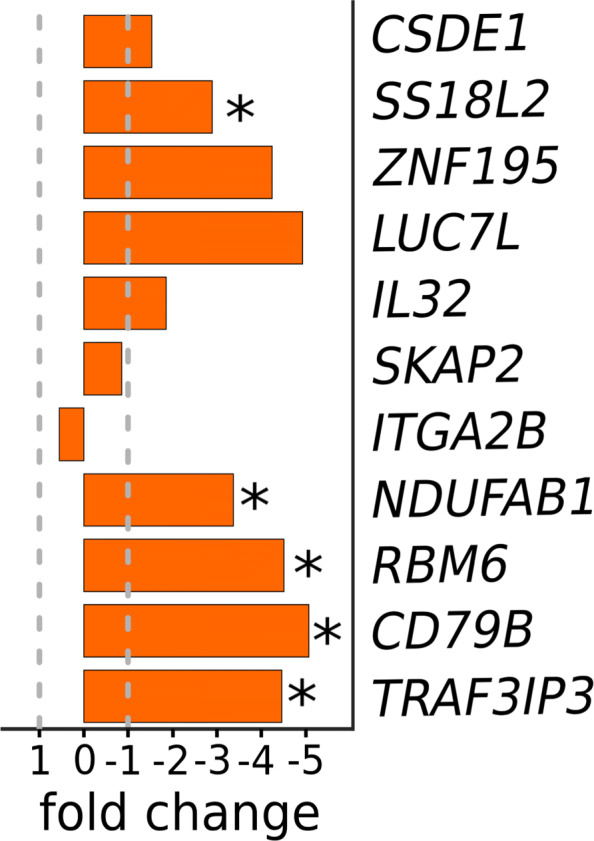
Results on validation dataset. Bar plots depicting the expression fold changes of the 11 genes between the healthy (n = 7) and NSCLC patients (n = 10). Asterisks represent *p*-value significance. *p*-value cutoff was set to 0.05. *, **, *** and **** represent the *p*-values of ≤0.05, ≤0.01, ≤0.001 and ≤0.0001 respectively

**Fig. 4:**
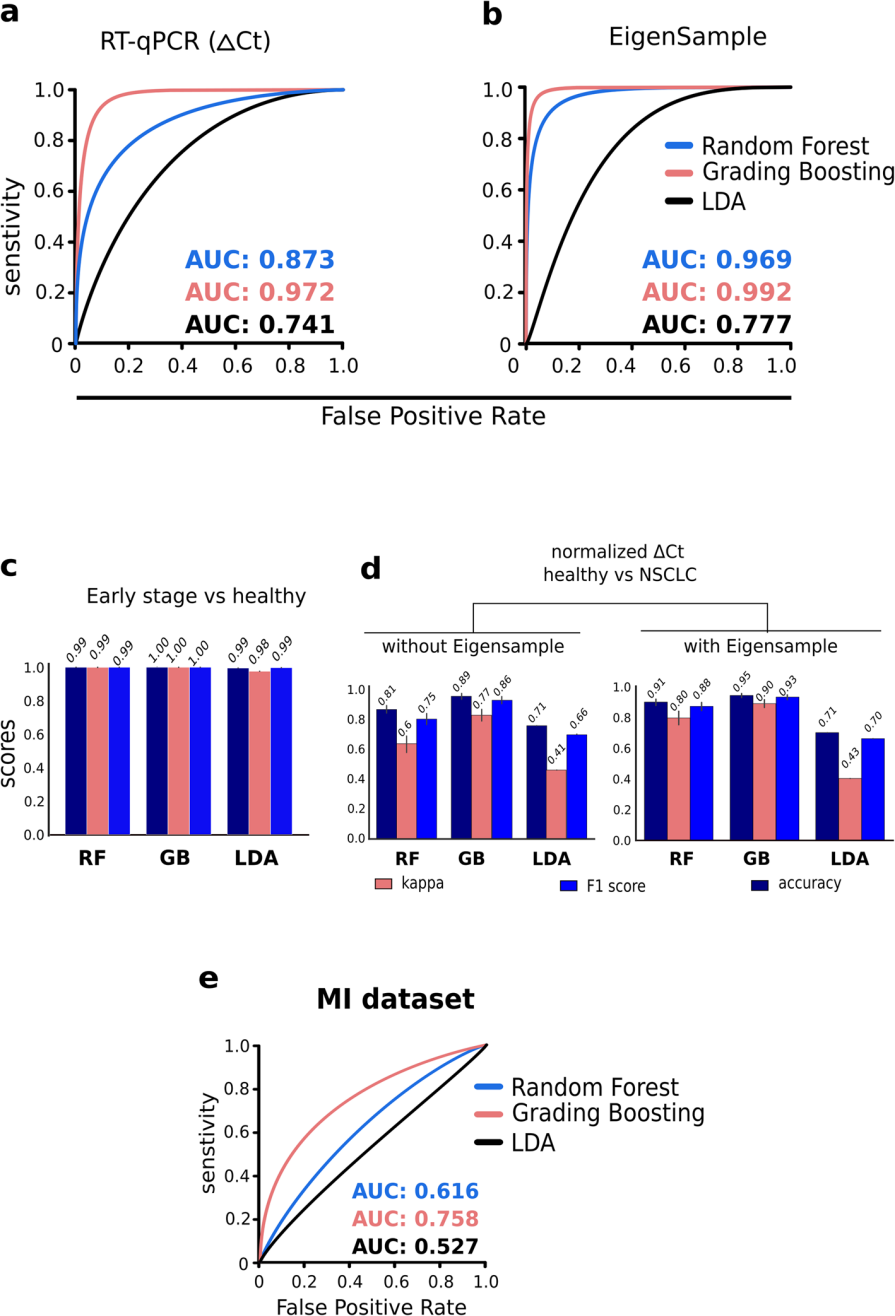
Performances of three independent classifiers on early-stage vs. healthy samples, MI samples and on RT-qPCR data. (**a**) AUC (Area under the curve) plot representing the performances of three independent classifiers i.e. Gradient Boosting Machines (GB), Random Forest (RF), and Linear Discriminant Analysis (LDA) in distinguishing tumor and healthy samples using *Δ* Ct values of 11 genes from 10 NSCLC patients and 7 healthy controls. (**b**) AUC plot depicting the improvement in the classification accuracy by augmenting the data-points with artificial samples, using the EigenSample technique. (**c**) Classification performance based on the proposed 11 gene-panel the on TEP profiles of non-metastatic NSCLC patients and healthy controls from [15]. (**d**) Classifier performances on experimental data of 10 NSLC and 7 healthy samples. **e** Receiver Operating Characteristics (ROC) plot depicting the performances of three independent classifiers in distinguishing healthy and myocardial infarction episode samples using normalized intensity from platelets microarray dataset [21]

We performed independent experimental validation of the panel to assert two major reproducibility concerns. The first concern was cross-geography reproducibility, and the second was cross-assay reproducibility. The RNA-Seq data we used for gene selection is representative of the Dutch population alone [4]. The universality of the gene panel could be probed only by reproducing its efficiency on a geographically distinct population. On the same line, it is equally important to check if the fidelity of the gene-panel remains intact with the change in the molecular assay. For instance, under many practical settings, RT-qPCR is more economically viable as compared to RNA-Seq. To this end, we used RT-qPCR to profile the expression of the selected 11 genes, on a cohort of 10 lung cancer patients (7 treat naive and 3 first-line chemotherapy) and 7 healthy controls (Fig. 2a - lower panel, Figure S1). Gene expression trends, observed in our RT-qPCR data (Fig. 3), were largely similar to that of the RNA-Seq study. Among the three classifiers, GB offered the highest accuracy (AUC = 0.97) (Fig. 4a, Table S2). RF and LDA offered AUC values of 0.87 and 0.74, respectively (Fig. 4a). To circumvent the paucity of RT-qPCR profiles, we employed EigenSample for producing artificial samples to augment the training data (refer Methods), which substantially enhanced the classifier performances with a maximum improvement of 10% (Table S2, Fig. 4b, d). With sample size augmentation, GB offered a staggering AUC of 0.99, for the RT-qPCR data (Fig. 4b). Best and colleagues [4] reported an accuracy of 96% for healthy vs NSCLC samples. On the same RNA-seq samples, the proposed 11 gene panel obtained 97% accuracy (Table S3). Xing and colleagues [17] studied and validated a single transcript, *ITGA2B* (present in our gene-panel), as a TEP marker for early stage NSCLC and obtained an AUC of 0.92. When we made classification models with *ITGA2B* alone, the highest AUC obtained was 0.78 on the pan-cancer dataset [4]. However, when we considered only non-metastatic NSCLC and healthy samples [15], the highest AUC was 0.95.

Our patient cohort primarily consisted of metastatic NSCLC samples (Table S4), due to the unavailability of early locally advanced cases. Best et al. [15] investigated TEPs on a larger cohorts of NSCLC patients and healthy samples (GSE89843). They reported an 81% accuracy for early locally advanced tumour classification using ∼1600 genes. We used the locally advanced and healthy samples from this study to test the applicability of our gene-panel in detecting the early onset of the disease. In this case, we hit an accuracy of 100%, indicating potential implementation of the panel in early cancer diagnosis (Fig. 4c). Of note, the 11 gene signature is devised for six different cancer types and validated on NSCLC samples. Survival analysis was performed independently on each of the 11 genes using the GEPIA (Gene Expression Profiling Interactive Analysis) web-server [18]. Based on the NSCLC cohort of TCGA, three of the 11 genes, namely *CD79B, NDUFAB1, TRAF3IP3* exhibited significantly divergent survival patterns across the high and low-risk groups (Figure S2).

### Cancer specificity of the gene signature

A significant factor that influences the success of a molecular screening test is its specificity. Changes in the molecular profile of platelets have already been reported in multiple disease conditions [4, 19, 20]. Cardiovascular diseases are prominent among these [19, 21, 22]. We asked if our gene panel is specific to cancers. To address this, we conducted a similar set of analyses on a distinct pathological condition, i.e. Myocardial Infarction (MI), where drastic shifts in the platelets transcriptome have been observed [21]. Since ST-segment Elevation Myocardial Infarction (STEMI) and Stable Coronary Artery Disease (SCAD) both cause perturbation in the platelet transcriptomes, samples with these conditions were grouped as one class (patients). The data, now having 2 classes (patient (*n*=38) vs healthy (*n*=19)), was then subjected to Leave-One-Out Cross-Validation (LOOCV) using 3 classifiers - RF, GB, LDA. Following the suite of NSCLC validation, RF and GB were run with 50 different seeds to estimate the stochasticity of the models. As expected, our 11-gene signature failed to discriminate between the healthy and the diseased specimens under equivalent experimental settings, thereby suggesting the specificity of the signature towards the tumour datasets (Table S2, Fig. 4e).

### Empanelled genes share their regulatory circuitries

**Fig. 5:**
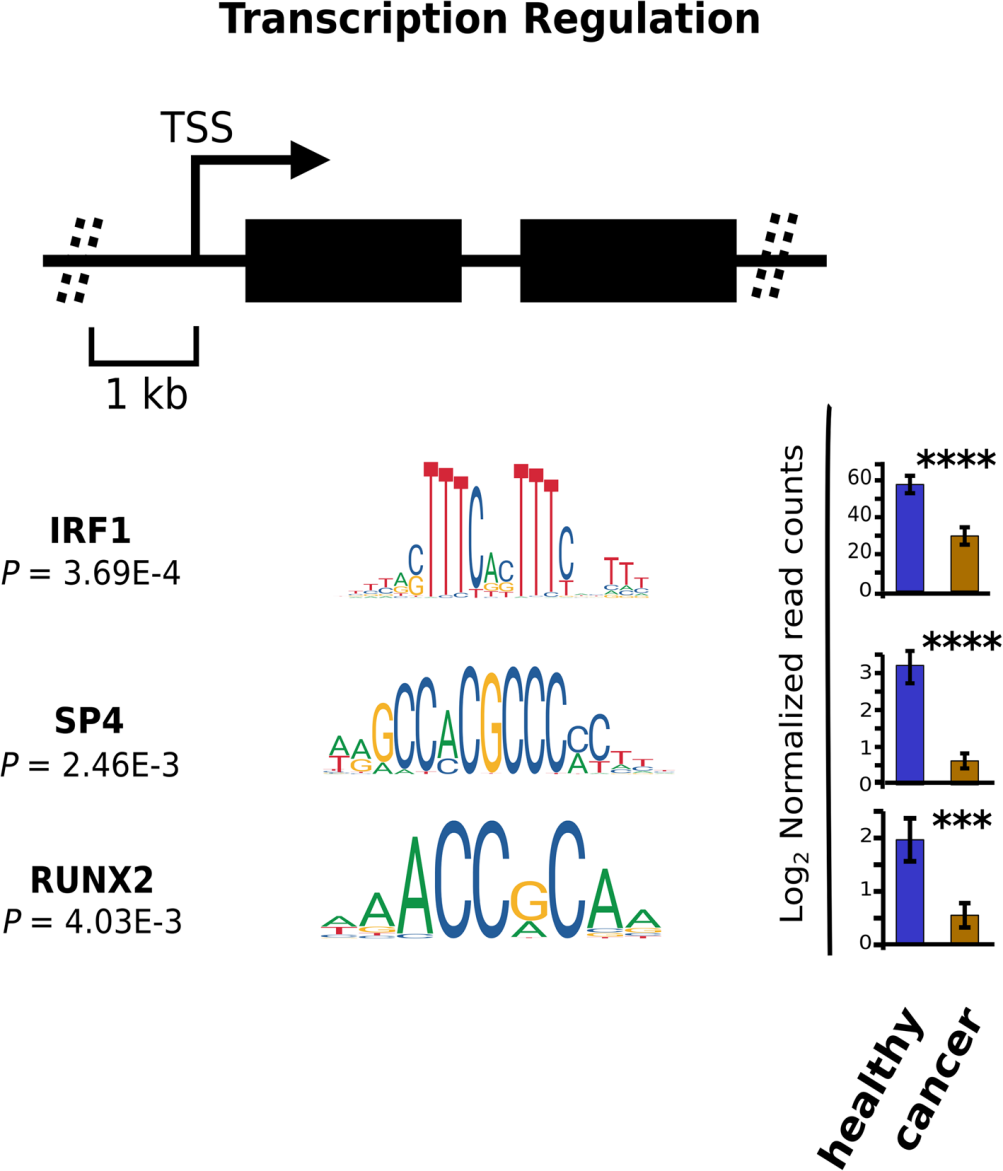
Gene panel shares a regulatory circuit. Graphical representation of the enriched transcription factor binding sites in the 1 kilobase upstream region (TSS=0) of 11 gene signature. *p*-value (FDR-corrected) represents the statistical power depicting a significant enrichment of the indicated motifs in the given region over shuffled control sequences. Bar graphs on the right represent normalized read-counts of the identified transcriptional factors between healthy and tumor samples. Asterisks represent *p*-value significance. *p*-value cutoff was set to 0.05. *, **, *** and **** represent the *p*-values of ≤0.05, ≤0.01, ≤0.001 and ≤0.0001 respectively

Our results using publicly available data [4] has shown the efficiency of our gene-panel (Fig. 1). Further, the RT-qPCR results concurred in terms of expression dynamics of the selected 11 genes, across cancer and control samples (Figs. 3, 4a,b,d). We conjectured that these genes could be co-regulated by a shared set of transcriptional factors (TFs). To check this, we scanned the putative promoter regions of all the genes for common transcription factor binding sites. For this, we extracted 1 kb upstream regions from the transcriptional start sites (TSS) of all the genes and scanned for transcription factor binding motifs. We could identify 3 potential transcriptional factors (*IRF1*, *SP4* and *RUNX2*) whose motifs were significantly enriched among the promoter sequences of the 11 genes (refer Methods). These TFs were all found to be downregulated in the NSCLC samples (Fig. 5). It should be noted that each of the three TFs, including their respective families, are well-reported in lung cancer literature [23–25]. These analyses, in combination with our RT-qPCR results, establish a potential regulatory link between these three transcription factors and the empanelled transcripts.

## Discussion

Platelets are long known for their role in linking tissue damage or malfunction with the inflammatory response [26, 27]. These megakaryocyte-derived anucleated cells interact significantly with cell types and release various factors [28]. Recent evidence hints at platelets’ involvement in cancer growth as well as metastasis [3, 4, 15, 29–31]. In cancer, platelet transcriptome undergoes significant changes, thereby providing a remarkable opportunity to utilize them in devising novel diagnostic strategies [3, 4, 15]. Problems with these approaches are two folds. First, an optimal prediction of the concerned disease often requires several tens of genes [4, 15]. Secondly, the validation of a gene signature is contingent on the availability of a large number of tissue samples [16]. To overcome these limitations, we developed a pipeline that maximizes disease-healthy classification performance with limited feature genes and small validation cohort. We successfully augmented the validation cohort with artificial data points, which further boosted the classification accuracy significantly. This could be really useful in a multitude of practical scenarios, where low sample acquisition rates impede the study progress and clinical adoption.

The 11 genes spotted by our workflow were validated on NSCLC cases and healthy controls at a near-perfect accuracy (Figs. 1, 4c). We found model accuracy to be consistent across both metastatic (Fig. 4d) as well as non-metastatic cases (Fig. 4c). A subset of the 11 gene signature has recently been reported in the context of lung cancer, either as an oncogenic driver (e.g. *CD79B* [32]) or a prognostic marker (e.g. *TRAF3IP3, SKAP2, and SS18L2*) [33–35]. Moreover, mutations in *RBM6* were associated with the loss of heterozygosity in the majority of lung cancer patients [36]. *ITGA2B* is a validated marker for the diagnosis of NSCLC using TEPs [17] with an AUC of 0.92. Differential expression of *IL-32* has been reported in various lung cancer histotypes, including small-cell lung cancers [37]. There are no reports which establish a direct association of the remaining four genes (*CSDE1, ZNF195, LUC7L, and NDUFAB1*) with NSCLC. Our survey identified that *CSDE1* is a validated target of the *C-MYC* transcription factor. *C-MYC* is a well-studied oncogene [38]. In the case of small lung-cancer cells, surprisingly, it harbors antagonistic function and suppresses the tumorigenicity [39]. Further, to establish a functional link between these 11 empanelled genes with NSCLC, we categorically identified their top 5 interaction partners at the protein level, using the STRING database [40]. Interrogation of the prominent interacting partners revealed their functional importance in lung cancer, indicating an indirect mode-of-association of these proteins with NSCLC (Table S5). The co-regulatory transcription factor analysis identified three core transcription factors (*IRF1*, *SP4* and *RUNX2*), which play potential roles in the regulation of the 11 empanelled genes. *IRF1*, *SP4* and *RUNX2* have been found to play an important role in megakaryocyte development and platelet production [41, 42].

All these collectively indicate that the dysregulation of some of these key platelet transcription factors might trigger a cascade of gene expression changes in TEPs w.r.t. the healthy platelets. Since all the key TFs are associated with platelet development, an expression study focusing on immature platelets can further our mechanistic understanding of the dysregulation of platelet transcriptomes in cancer. In line with this, the quantitative estimation of the key morphometric features of developed/developing platelets could be substantially insightful.

The gene-panel was found to be non-decisive on platelet transcriptomes collected from patients with Myocardial Infarction (MI) (Fig. 4e, Table S2), which asserts its specificity to cancer. In view of the encouraging results discussed in this study, we believe the proposed gene panel will attract further validation and clinical adoption.

In this article, we demonstrated the predictive power of a small set of platelet genes in determining the existence of cancer. Similar strategies can be developed for inferring the potential cancer types. In all these cases, the gene panels need to be validated on larger patient and control samples’ cohorts. An orthogonal application of such panels could be tracking the treatment responses, as well as the recurrence of the disease.

## Conclusion

Liquid biopsy, a powerful and non-invasive method for early diagnosis of cancer, is reforming the field of clinical diagnostics. We proposed an 11 platelet-gene panel (*CD79B, CSDE1, IL-32, ITGA2B, LUC7L, NDUFAB1, RBM6, SKAP2, SS18L2, TRAF3IP3, and ZNF195*) that provides reliable and economically viable platelet-based classification between cancer and healthy samples. The gene-panel can accurately diagnose both early and late-stage NSCLC cases. We performed validation of the gene panel on two independent cohorts of NSCLC patients, of which one belonged to the present study. These cohorts feature a total of 67 NSCLC patients representing both early and late-stage of cancer. For the published and in-house datasets, we attained an AUC of 1 and 0.99, respectively, using the Gradient Boosting Machines (GB) classification algorithm. These, by far, outwit the published classification accuracies, wherein the number of genes used by the models is significantly higher (approximately 1000 genes). Notably, we found the genes to share transcription factor binding motifs, recognized by a small number of transcription factors (TFs), namely *IRF1, SP4,* and *RUNX2*. We also determined the cancer-specificity of the gene-panel by benchmarking their performance on a platelet-based MI dataset.

## Methods

### Datasets

Best and colleagues performed RNA sequencing of platelets collected from cancer patients and healthy individuals (Accession ID: GSE68086) [4]. From this study, we obtained 273 TEP expression profiles spanning six cancer types: non-small-cell lung cancer (NSCLC): 59, colorectal cancer (CRC): 44, glioblastoma multiforme (GBM): 40, breast cancer (BRCA): 38, pancreatic cancer (PC): 33, hepatobiliary cancer (HBC): 5. In addition to the cancer samples, platelets from 54 healthy individuals were also profiled. The dataset originally had 283 samples. We filtered out samples (*n*=10) with unknown labels, and low expression count. We also used TEP expression profiles from non-metastatic NSCLC cases and healthy samples from an independent study (GEO Accession ID: GSE89843) [15], as a test cohort. Gene expression profiles (raw read counts) were normalized using the TMM normalization method (edgeR package) [43].

In order to examine the gene panel’s ability to classify early-stage cancer, we selected platelet RNA-seq samples consisting of 57 early locally advanced NSCLC patients (non-metastatic) and 377 healthy individuals from an independent study by [15] (GSE89843).

To assess the specificity of our gene-panel, we re-analyzed platelet transcriptomes from patients with Myocardial Infarction (MI) (GEO Accession ID: GSE109048) [21]. The dataset consisted of microarray gene expression profiles, obtained from 57 platelet samples with the following distribution: 19 ST-segment Elevation Myocardial Infarction (STEMI), 19 patients with Stable Coronary Artery Disease (SCAD), and 19 healthy donors. SCAD and STEMI are both phenotypically similar conditions and have been shown to cause changes in platelet gene expression [16]).

### Gene selection

We evaluated several supervised and unsupervised gene selection strategies, namely Wilcoxon rank-sum test [44], Logistic regression [45], Coefficient of Variance (CV) [46], Analysis of Variance (ANOVA) [47], minimum redundancy maximum relevance (MRMR) [48], and DESeq2 [49]. Intending to discover a frugal gene panel, we selected up to a maximum of 15 genes for each case. We also evaluated the combinations of the supervised and unsupervised techniques and found out that CV-ANOVA combination yields the most favorable outcome (Table S6). At first, we selected 1000 most variable genes based on the Coefficient of Variance (CV), which is an unsupervised method. Independently, we selected the top 1000 genes based on differential expression tests conducted using ANOVA. We obtained a set of 11 genes upon intersecting the results from these two approaches. All these techniques were applied to identify genes, which could distinguish between the samples from six cancer types and the healthy controls as reported by Best and colleagues [4]. The CV-ANOVA intersection based approach offered a total of 11 genes namely *CD79B, CSDE1, IL-32, ITGA2B, LUC7L, NDUFAB1, RBM6, SKAP2, SS18L2, TRAF3IP3, and ZNF195*. The workflow is outlined in (Fig. 2a). We validated the gene panel on each of the cancer subtypes and found NSCLC and breast cancer to have the highest accuracies (Table S3). Because of the highest statistical confidence, we performed all the downstream analyses with NSCLC, including its experimental validations.

### Validation of the gene panel on RNA-Seq data

We used the selected genes to train classification models using three widely used techniques, namely Gradient Boosting Machines (GB) [50], Random Forest (RF) [51], and Linear Discriminant Analysis (LDA) [52]. To do this, we utilized the RNA-Seq read count data from a study by Best and colleagues [4]. As a benchmark, we considered comparing our predictions with ones obtained using 1000 genes that the authors proposed. We created 100 sets of 90-10 train-test stratified splits of the data for the area under the curve (AUC) measurements (Fig. 1, Table S1). We also checked the performance of the 11 genes using only NSCLC (*n*=59) and healthy samples (*n*=54) (Figure S3). To gauge the predictive power of the gene-panel for early cancer diagnosis, we chose non-metastatic NSCLC samples and healthy samples from [15]. To benchmark our findings against the reported values, we performed Leave-One-Out Cross-Validation (LOOCV) in tune with the methodology followed by Best et al. [15]. LOOCV for each classifier was performed over 50 times with random seeds to measure the volatility of the models.

### Clinical samples

Blood samples were collected from a total of 10 NSCLC patients and 7 healthy subjects (control) to train classifiers on data generated from the RT-qPCR experiment for validation purposes. We obtained ethical clearance from the Institute Ethics Committee at the All India Institute of Medical Sciences-New Delhi. All donors provided informed consent before the collection of peripheral blood. 15 ml of peripheral blood was collected in a BD Vacutainer tube containing anticoagulant EDTA. The experimental workflow is outlined in Figure S1. Clinical information about cancer patients is summarised in Table S4.

### Platelets isolation from whole blood

The platelet-rich plasma (PRP) fraction was prepared by centrifugation of whole blood for 20 minutes at 120 x g at room temperature. The supernatant (PRP) was transferred into a fresh vial, and the red blood cell pellet was discarded after the first round of centrifugation. The platelets were enriched from PRP by centrifugation at 360 x g for 20 min at room temperature. The pellet representing platelets was washed with 1X Phosphate Buffered Saline (PBS) and centrifuged at 5000 rpm for 5 min. The PBS was discarded, and platelet pellet was re-suspended in 1*ml* TRI reagent ^®^ (SIGMA-Aldrich, USA) and stored at -80^∘^C.

### RNA isolation from platelets

We performed total RNA isolation as per the manufacturer’s recommendations (TRI reagent (SIGMA-Aldrich, USA)). Samples in the TRI reagent ^®^ were thawed and mixed with 200 *μl* chloroform. After vigorous shaking, the samples were incubated for 15 min at room temperature, followed by centrifugation at 12000 x g for 15 min at 4^∘^C. The aqueous layer was carefully transferred into fresh vials, and 500 *μl* of isopropanol was added for RNA precipitation. After incubation for 10 min at room temperature, samples were centrifuged at 12000 x g for 10 min at 4^∘^C. Next, we discarded the supernatant, and washed the RNA pellet twice with 75% ethanol, followed by centrifugation at 7500 x g for 5 min. After centrifugation, the RNA pellets were dried at room temperature and resuspended in 30 *μl* RNase-free water. RNA samples were quantitated using Nanodrop and stored at -80^∘^C. cDNA synthesis was performed using the standard protocol as provided by the manufacturer cDNA kit (cat no. K1622, Thermo Fisher Scientific, USA). Briefly, the reaction mixture for cDNA synthesis was setup with 4 *μl* 5X buffer, 2 *μl* dNTPs, 1 *μl* Random primer (RP), 1 *μl* RiboLock (RL) and 1 *μl* SuperScript Reverse Transcriptase and RNA sample in a total of 20 *μl* volume.

### Experimental validation of the gene panel using RT-qPCR

TaqMan gene expression assays (Applied Biosystems, California, USA) were used for expression studies of shortlisted gene candidates, namely *CD79B, CSDE1, IL-32, ITGA2B, LUC7L, NDUFAB1, RBM6, SKAP2, SS18L2, TRAF3IP3, and ZNF195.* Two reference genes (*ACTB* and *GAPDH*) were used as internal controls for downstream normalization steps. The reaction mix was prepared using 10 *μl* Master mix, 1 *μl* of gene expression assay, Nuclease-free water and cDNA sample per well.

### Preprocessing of the RT-qPCR data

For the estimation of the relative gene expression, we used the comparative-Ct (*ΔΔ*Ct) method [53]. By using this approach, we first normalised our data using reference genes and then calculated the relative expression differences for each gene (healthy vs cancer) by fold-change. Two different reference genes *ACTB* and *GAPDH*) were used for expression normalisation. We modelled our calculations and statistical analysis based on previously published examples [53–55].

### EigenSample based artificial augmentation of the validation cohort

The EigenSample technique [56] was employed to augment the training data as subsampled from the entire set of RT-qPCR profiles. EigenSample fabricates artificial data points in a manner that least perturbs the variance of the original dataset. First of all, it projects the input data on a small number of principal components. Class labels are then used to define clusters, whose centres are joined to the samples of the respective classes by straight lines. Midpoints of these straight lines are now considered as new samples. Each new sample *x*^*i*^ in the lower dimension is projected back to pre-images *z*^*i*^ in the original dimension by solving a quadratic programming problem that respects the minimum and maximum bounds of the original training data (Eqs. 1 to 5). Earlier experiments with EigenSample have shown that the new samples it generates more realistic and authentic than other methods. Let *P* be the projection matrix that transforms a high dimensional sample *z* to a low dimensional image *x*. The pre-image of a new sample *x*^*i*^ is denoted by *z*^*i*^ and is obtained by solving the following optimization problem.

1 $$\begin{array}{@{}rcl@{}} \underset{z^{i},q^{i+},q^{i-}}{\text{Minimize}} &\frac{1}{2} \|z^{i}\|^{2} + C \sum_{j= 1}^{k} \left(q_{j}^{i+} + q_{j}^{i^{-}}\right)  \end{array} $$

2 $$\begin{array}{@{}rcl@{}} & \text{ s.t.}  \\ & P \cdot z^{i} - q^{i^{+}} \leq x^{i} + \epsilon  \end{array} $$

3 $$\begin{array}{@{}rcl@{}} & P \cdot z^{i} + q^{i^{-}} \geq x^{i} - \epsilon  \end{array} $$

4 $$\begin{array}{@{}rcl@{}} & lb \leq z^{i} \leq ub  \end{array} $$

5 $$\begin{array}{@{}rcl@{}} & q^{i+}, q^{i-} \geq 0  \end{array} $$

where *ε* is the approximation tolerance, and *q*^*i*+^ and *q*^*i*−^ are error variables. *C* is a hyper-parameter controlling the trade-off between the degree of approximation and norm of the solution vector ∥*z*^*i*^∥. A small value of *C* ($C \xrightarrow {} 0$) will yield a minimum norm solution, while large *C* ($C \xrightarrow {} \infty $) corresponds to the solution of a system of linear equations. Deploying machine-learning techniques on small sample sizes is difficult. Data augmentation is an important tool to increase the size of the labelled data and helps us to use the existing data more effectively [57, 58]. As such, we used EigenSample to demonstrate that artificial augmentation of training data can improve the prediction outcomes.

### Validation of the gene panel on RT-qPCR data

Due to the small sample size, we resorted to the Leave-One-Out Cross-Validation (LOOCV) strategy for assessing the performance of various classifiers on RT-qPCR data. On every pass of LOOCV, we applied EigenSample for training-data augmentation. For RF and GB classifiers, 50 random seeds were used to control their inherent stochasticity. The ROC plot was constructed while pooling predictions across these runs.

### Exploring the co-regulatory network of the selected genes

To identify the potential transcription factors (TFs), regulating the empanelled genes, we extracted their putative promoter regions (1kb upstream of the transcriptional start site; TSS) using Eukaryotic Promoter Database [59]. Promoter sequences thus obtained were converted into FASTA format and were subjected to the Analysis of Motif Enrichment (AME) tool (a feature of the MEME suite), to discover common TF binding motifs [60]. For accurate inference of the common transcription factor binding sites (TFBSs) in the promoter sequences, we have utilized JASPER motif database [61], a reliable database harboring non-redundant transcription factor (TF)-binding profiles. Enrichment analysis of the common regulatory transcription factors was performed against randomly shuffled input sequences (control sequences). Fisher’s exact test was used to report *p*-values. Differential expression of the TFs in the RNA-seq data [15] was calculated using edgeR [62] (Table S7). Only NSCLC and healthy samples were selected from the dataset for the analysis.

## Supplementary information

**Additional file 1** Supplementary Data: Meta-analysis of Tumor-Educated-Platelet transcriptomes reveals a concise molecular signature for blood-based detection of early and late NSCLC

## Data Availability

The RT-qPCR dataset generated for this study are available on request to the corresponding author.
