# Correction to: Molecular signature comprising 11 platelet-genes enables accurate blood-based diagnosis of NSCLC

**DOI:** 10.1186/s12864-020-07230-5

**Published:** 2020-12-08

**Authors:** Chitrita Goswami, Smriti Chawla, Deepshi Thakral, Himanshu Pant, Pramod Verma, Prabhat Singh Malik, Jayadeva, Ritu Gupta, Gaurav Ahuja, Debarka Sengupta

**Affiliations:** 1 Department of Computer Science and Engineering, Indraprastha Institute of Information Technology, New Delhi, India; 2 Department of Computational Biology, Indraprastha Institute of Information Technology, New Delhi, India; 3 Laboratory Oncology Unit, All India Institute of Medical Sciences, New Delhi, India; 4 Department of Electrical Engineering, Indian Institute of Technology, New Delhi, India; 5 Department of Medical Oncology, All India Institute of Medical Sciences, New Delhi, India; 6 Institute of Health and Biomedical Innovation, Queensland University of Technology, Brisbane, Australia; 7 Centre for Artificial Intelligence, Indraprastha Institute of Information Technology, New Delhi, India

**Correction to: BMC Genomics 21, 744 (2020)**

**https://doi.org/10.1186/s12864-020-07147-z**

Following the publication of the original article [1], it was reported that Ritu Gupta was not listed as a corresponding author in the online version of the article. The original article has been corrected.
