# Supplementary material for: Molecular signature comprising 11 platelet-genes enables accurate blood-based diagnosis of NSCLC
Source: BMC Genomics. 2020 Oct 27;21:744. doi: 10.1186/s12864-020-07147-z (PMC7590669; doi:10.1186/s12864-020-07147-z)
Supplement: Supplementary file 1 — Additional file 1 Supplementary Data: Meta-analysis of Tumor-Educated-Platelet transcriptomes reveals a concise molecular signature for blood-based detection of early and late NSCLC [file 12864_2020_7147_MOESM1_ESM.pdf]

Supplementary Data: Meta-analysis of Tumor-Educated-Platelet transcriptomes reveals a concise molecular signature for blood-based detection of early and late NSCLC

Chitrita Goswami[1‡], Smriti Chawla[2‡], Deepshi Thakral[3], Himanshu Pant[4], Pramod Verma[3], Prabhat Singh Malik[5], Jayadeva[4], Ritu Gupta[3]*, Gaurav Ahuja[2]*, and Debarka Sengupta[1,2,6,7]*

[1]Department of Computer Science and Engineering, Indraprastha Institute of Information Technology, Delhi

[2]Department of Computational Biology, Indraprastha Institute of Information Technology, Delhi

[3]Laboratory Oncology Unit, All India Institute of Medical sciences, Delhi

[4]Department of Electrical Engineering, Indian Institute of Technology, Delhi

[5]Department of Medical Oncology, All India Institute of Medical sciences, Delhi

[6]Institute of Health and Biomedical Innovation, Queensland University of Technology, Brisbane, Australia.

[7]Centre for Artificial Intelligence, Indraprastha Institute of Information Technology, New Delhi, India

To whom correspondence should be addressed. Debarka Sengupta; Tel: (+91)11-26907446; Email: debarka@iiitd.ac.in. Correspondence may also be addressed to: Ritu Gupta; Tel: (+91)112659443; Email: drritugupta@gmail.com and Gaurav Ahuja; Tel: (+91)11-26907475; Email: gaurav.ahuja@iiitd.ac.in.

Supplementary Tables

Table S1: Table representing the comparative performance of three different classifiers with 1000 and 11 gene signatures.

**RF**	Gene Signature: 1000	11
Accuracy	0.89	0.87
F1 score	0.74	0.68
AUC	0.94	0.93
Kappa	0.52	0.52

**GB**	Gene Signature: 1000	11
Accuracy	0.89	0.89
F1 score	0.69	0.69
AUC	0.90	0.94
Kappa	0.61	0.62

**LDA**	Gene Signature: 1000	11
Accuracy	0.92	0.89
F1 score	0.80	0.72
AUC	0.94	0.91
Kappa	0.63	0.60

Table S2: Table representing the comparative performance of three different classifiers, with and without EigenSampling of the Cq values of the 11 gene signature inferred from qPCR. The table also contains the performance of these classifiers on the Myocardial Infarction dataset.

	Eigensample		
	without	with	
**RF**	ΔCT values		MI samples
Accuracy	0.81	0.91	0.52
F1 score	0.75	0.88	0.18
AUC	0.87	0.97	0.61
Kappa	0.60	0.80	-0.15

	Eigensample		
	without	with	
**GB**	ΔCT values		MI samples
Accuracy	0.89	0.95	0.53
F1 score	0.86	0.93	0.00
AUC	0.97	0.99	0.75
Kappa	0.77	0.90	-0.22

	Eigensample		
	without	with	
**LDA**	ΔCT values		MI samples
Accuracy	0.71	0.71	0.57
F1 score	0.66	0.70	0.29
AUC	0.74	0.77	0.61
Kappa	0.41	0.43	0.00

Table S3: Table representing the comparative performance of three different classifiers to classify each cancer subtype vs healthy cohort.

Cancer type vs Healthy		RF	GB	LDA
NSCLC	Accuracy	0.97	0.94	0.97
	F1 score	0.68	0.69	0.72
	AUC	0.96	0.92	0.96
	Kappa	0.70	0.69	0.71
BRCA	Accuracy	0.93	0.95	0.91
	F1 score	0.61	0.65	0.64
	AUC	0.94	0.93	0.92
	Kappa	0.67	0.68	0.65
CRC	Accuracy	0.91	0.90	0.90
	F1 score	0.58	0.56	0.54
	AUC	0.90	0.89	0.88
	Kappa	0.62	0.63	0.63
GBM	Accuracy	0.81	0.82	0.76
	F1 score	0.59	0.62	0.61
	AUC	0.87	0.84	0.77
	Kappa	0.66	0.57	0.52
PC	Accuracy	0.88	0.86	0.85
	F1 score	0.66	0.58	0.51
	AUC	0.85	0.81	0.80
	Kappa	0.59	0.59	0.53
HBC	Accuracy	0.57	0.59	0.58
	F1 score	0.35	0.42	0.10
	AUC	0.52	0.55	0.50
	Kappa	0.20	0.24	0.25

Table S4: Distribution of healthy controls and NSCLC samples according to their treatment status and cancer stage

Samples			Count
Treatment naive	7		10
First line chemotherapy	3		
Healthy	7		7
Stage-wise count of NSLC samples			
AJCC stage	Count	TNM Stage	Count
IIIA	1	T2N1M1a	1
IIIB	2	T3N3M0	1
IIIC	1	T4N0M0	3
IVA	4	T4N3M1b	3
IVB	2	T4N2M1c	2

Table S5: STRING network of top 5 interactants of the proteins associated with each of the 11 genes along with their roles in NSCLC.

**Protein**	**STRING reported associations**	**NSCLC links**
SKAP2	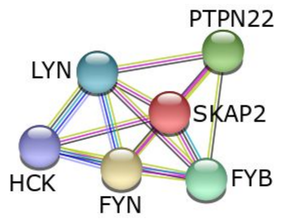	Proteins in the network that play a role in lung cancer - LYN [1, 2], HCK [3], FYN [4].
TRAF3IP3	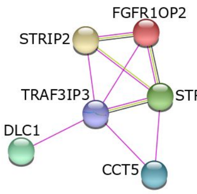	Proteins in the network that play a role in lung cancer - STRIP2 [5], STRN [6], DLC1 [7], CCT5 [8].
RBM6	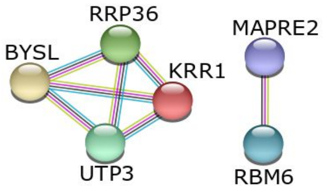	Proteins in the network that play a role in lung cancer - MAPRE2 [9].
ITGA2B	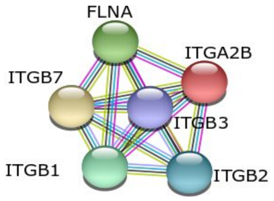	Proteins in the network that play a role in lung cancer - FLNA [10], ITGB1 [11], ITGB3 [12, 13].
IL32	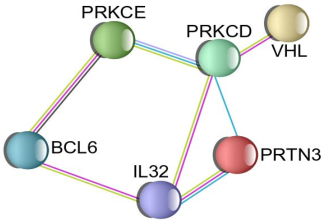	Proteins in the network that play a role in lung cancer - BCL6 [16], PRKCE [17], PRKCD [18]
CD79B	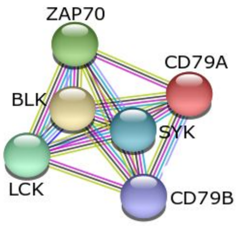	Proteins in the network that play a role in lung cancer - LCK [23], SYK [24], CD79A [25]

NDUFAB1	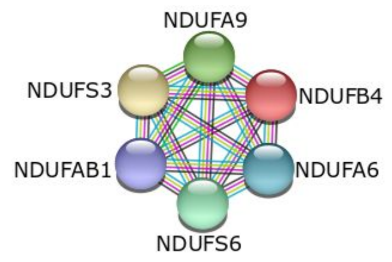	Proteins in the network that play a role in lung cancer - NDUFS3, NDUFS6, NDUFA6, NDUFB4, NDUFA9 [29]
LUC7L	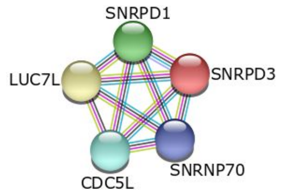	Proteins in the network that play a role in lung cancer - CDC5L [31], SNRPD3 [32].
ZNF195	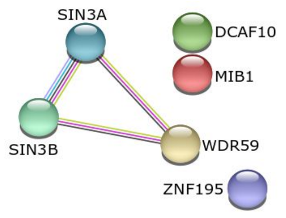	Proteins in the network that play a role in lung cancer -<br>SIN3A [33, 34], DCAF10 [35], MIB1 [36]
SS18L2	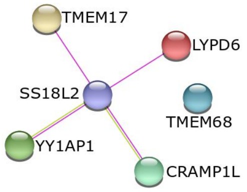	Proteins in the network that play a role in lung cancer - TMEM17 [37].
CSDE1	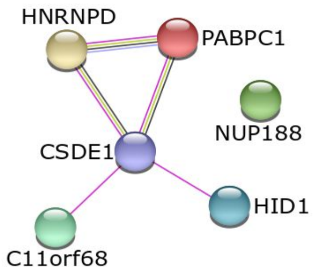	Proteins in the network that play a role in lung cancer - HNRNPD [38]

Note: Description of the different colour lines:

- Red line - indicates the presence of fusion evidence
- Green line - neighborhood evidence
- Blue line - cooccurrence evidence
- Purple line - experimental evidence
- Yellow line - text mining evidence
- Light blue line - database evidence
- Black line - coexpression evidence

Table S6: Table representing the comparative performance of different feature selection methods against the three different classifiers

Feature selection		RF	GB	LDA
MRMR	Accuracy	0.87	0.81	0.81
	F1 score	0.61	0.57	0.49
	AUC	0.80	0.62	0.63
	Kappa	0.50	0.43	0.44
Wilcoxon	Accuracy	0.83	0.89	0.85
	F1 score	0.56	0.59	0.54
	AUC	0.76	0.81	0.77
	Kappa	0.42	0.53	0.47
DESeq2	Accuracy	0.87	0.86	0.88
	F1 score	0.57	0.56	0.62
	AUC	0.81	0.80	0.82
	Kappa	0.45	0.44	0.51
CV	Accuracy	0.81	0.82	0.76
	F1 score	0.54	0.54	0.70
	AUC	0.62	0.61	0.81
	Kappa	0.40	0.41	0.49
ANOVA	Accuracy	0.83	0.6	0.78
	F1 score	0.56	0.47	0.49
	AUC	0.77	0.52	0.60
	Kappa	0.43	0.36	0.41
Logistic Regression	Accuracy	0.82	0.83	0.78
	F1 score	0.60	0.61	0.53
	AUC	0.67	0.67	0.65
	Kappa	0.45	0.44	0.37
CV and ANOVA	Accuracy	0.87	0.89	0.89
	F1 score	0.68	0.69	0.72
	AUC	0.93	0.94	0.91
	Kappa	0.52	0.62	0.61

Table S7: *P*-values associated with differential expression of transcription factors using different techniques.

	DESeq2	edgeR	Limma Voom
MEF2C	2.00E-06	0.19	0.13
IRF1	3.96E-06	3.88E-05	1.49E-07
SP4	2.29E-06	1.55E-07	4.50E-09
RUNX2	1.03E-03	6.1E-04	1.79E-07
SP2	0.02	0.58	0.02

Note : We extracted 1 kb upstream regions from the transcriptional start sites (TSS) of all the genes and scanned for transcription factor binding motifs. We found 5 common TFs (MEF2C, IRF1, SP4,, RUNX2 and SP2). We used three three widely used methods, namely DESeq2 [41], edgeR [42] and Limma Voom [43] to calculate their differential expression. We retained three of the five TFs (IRF1, SP4, RUNX2), which were observed to be significant across all three methods. For simplicity, have utilized the P-values obtained from edgeR.

Supplementary Figures

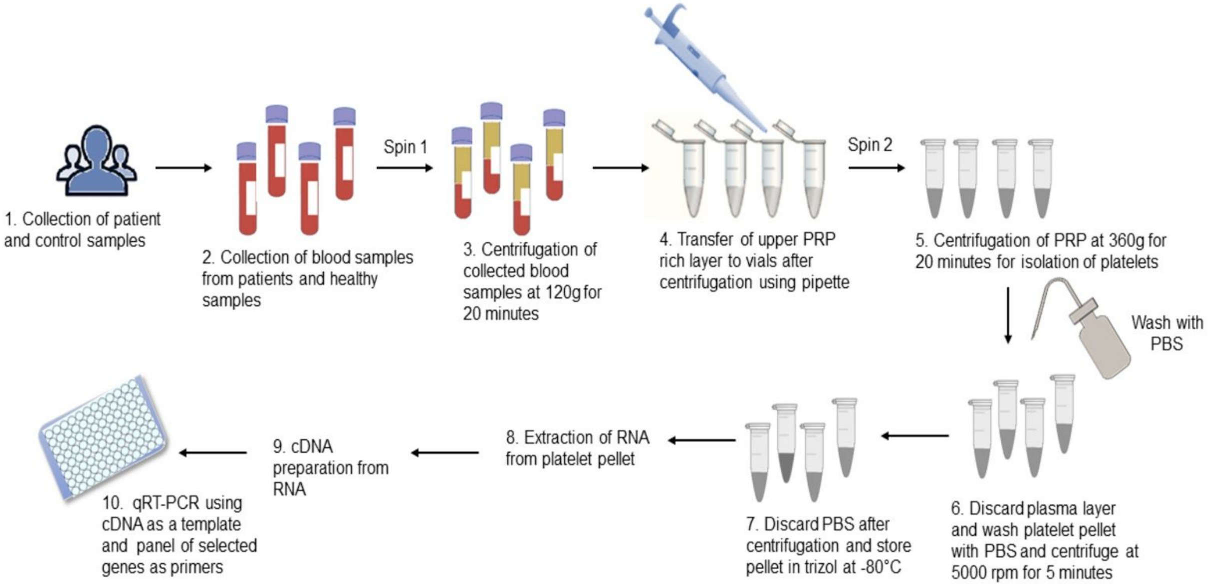

Figure S1: Schematic representation describing the key steps of the experimental workflow, involving selective isolation of the blood platelets, its total RNA isolation, complementary DNA preparation, and qPCR, employed in the validation of the 11 platelet-gene panel.

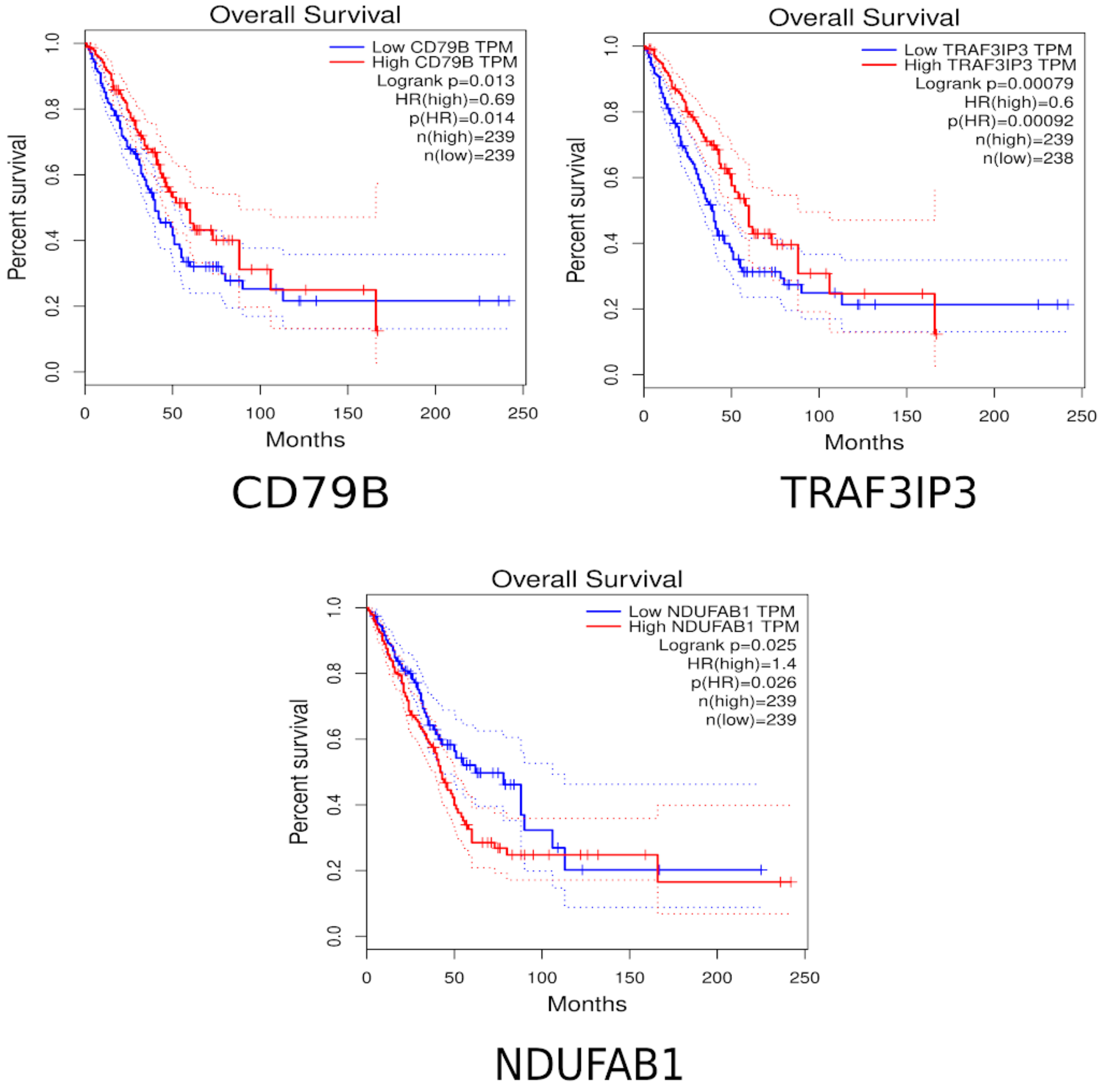

Figure S2: Survival plots depicting a section of the empanelled genes as prognostic markers for lung cancer.

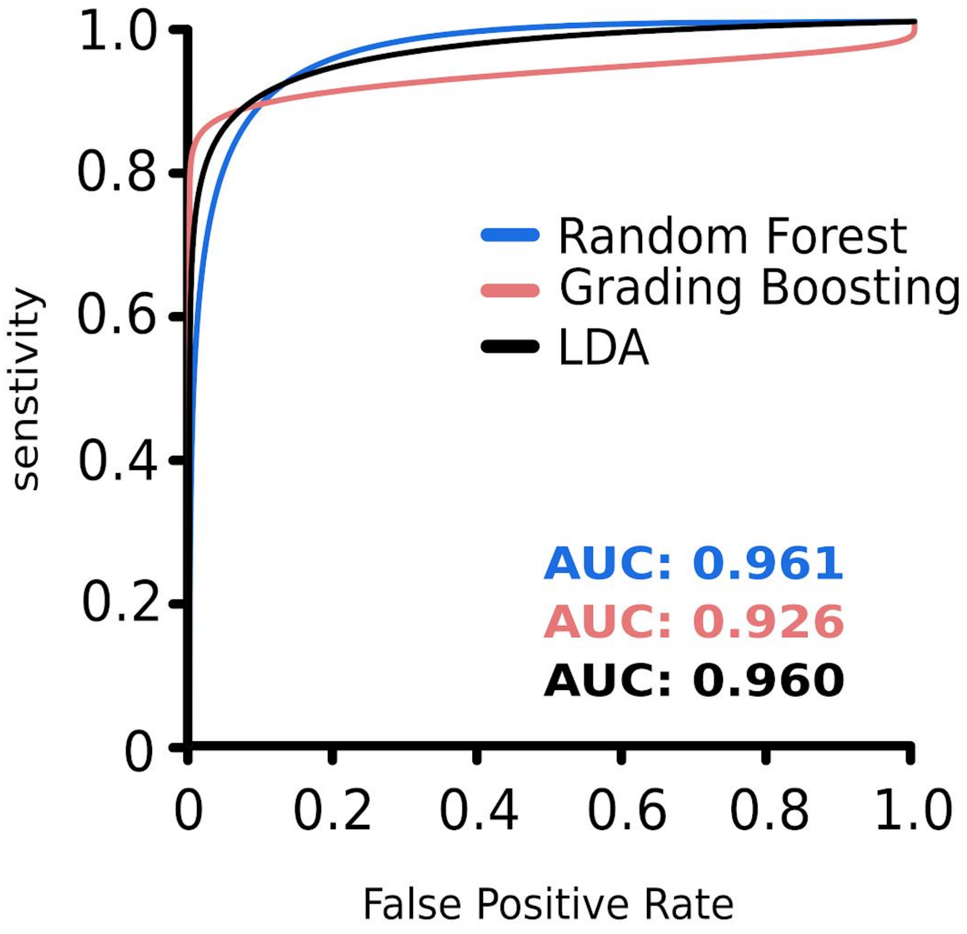

Figure S3: Receiver Operating Characteristics (ROC) plot representing the performances of three independent classifiers i.e. Gradient Boosting Machines (GB), Random Forest (RF), and Linear Discriminant Analysis (LDA) using normalized read counts of 11 genes from Tumor Educated Platelets transcriptome dataset. This cohort contains expression profiles of 59 NSCLC patients and 54 healthy subjects from GSE68086.

References

1. Kim YJ, Sung M, Park MJ, Shin YK, Choi Y-L. Abstract 2785: LYN is a new prognostic and therapeutic target in non-small cell lung cancer. Cancer Res. 2014;74 19 Supplement:2785–2785. doi:10.1158/1538-7445.AM2014-2785.

2. Kim YJ, Hong S, Sung M, Park MJ, Jung K, Noh K-W, et al. LYN expression predicts the response to dasatinib in a subpopulation of lung adenocarcinoma patients. Oncotarget. 2016;7:82876–88. doi:10.18632/oncotarget.12657.

3. Poh AR, O'Donoghue RJJ, Ernst M. Hematopoietic cell kinase (HCK) as a therapeutic target in immune and cancer cells. Oncotarget. 2015;6:15752–71. doi:10.18632/oncotarget.4199.

4. Zhou L-N, Li S-C, Li X-Y, Ge H, Li H-M. Identification of differential protein-coding gene expressions in early phase lung adenocarcinoma. Thorac Cancer. 2018;9:234–40. doi:10.1111/1759-7714.12569.

5. Qiu L-M, Sun Y-H, Chen T-T, Chen J-J, Ma H-T. STRIP2, a member of the striatin-interacting phosphatase and kinase complex, is implicated in lung adenocarcinoma cell growth and migration. FEBS Open Bio. 2020;10:351–61. doi:10.1002/2211-5463.12785.

6. Yang Y, Qin S-K, Zhu J, Wang R, Li Y-M, Xie Z-Y, et al. A Rare STRN-ALK Fusion in Lung Adenocarcinoma Identified Using Next-Generation Sequencing-Based Circulating Tumor DNA Profiling Exhibits Excellent Response to Crizotinib. Mayo Clin Proc Innov Qual Outcomes. 2017;1:111–6. doi:10.1016/j.mayocpiqo.2017.04.003.

7. Healy KD, Hodgson L, Kim T-Y, Shutes A, Maddileti S, Juliano RL, et al. DLC-1 suppresses non-small cell lung cancer growth and invasion by RhoGAP-dependent and independent mechanisms. Mol Carcinog. 2008;47:326–37. doi:10.1002/mc.20389.

8. Gao H, Zheng M, Sun S, Wang H, Yue Z, Zhu Y, et al. Chaperonin containing TCP1 subunit 5 is a tumor associated antigen of non-small cell lung cancer. Oncotarget. 2017;5:64170–9. doi:10.18632/oncotarget.19369.

9. Tran Q-N. A novel method for finding non-small cell lung cancer diagnosis biomarkers. BMC Med Genomics. 2013;6 Suppl 1:S11. doi:10.1186/1755-8794-6-S1-S11.

10. Zhang Y, Zhu T, Liu J, Liu J, Gao D, Su T, et al. FLNa negatively regulated proliferation and metastasis in lung adenocarcinoma A549 cells via suppression of EGFR. Acta Biochim Biophys Sin (Shanghai). 2018;50:164–70. doi:10.1093/abbs/gmx135.

11. Lai T, Hsiao M. ITGB1-DT and ITGB1 enhance lung cancer cell metastatic abilities and are associated with poor prognoses in NSCLC patients. The FASEB Journal. 2019.

12. Ni R, Huang Y, Wang J. miR-98 targets ITGB3 to inhibit proliferation, migration, and invasion of non-small-cell lung cancer. Onco Targets Ther. 2015;8:2689–97. doi:10.2147/OTT.S90998.

13. Zhu C, Kong Z, Wang B, Cheng W, Wu A, Meng X. ITGB3/CD61: a hub modulator and target in the tumor microenvironment. Am J Transl Res. 2019;11:7195–208.

14. Bronte G, Ulivi P, Verlicchi A, Cravero P, Delmonte A, Crinò L. Targeting RET-rearranged non-small-cell lung cancer: future prospects. Lung Cancer (Auckl). 2019;10:27–36. doi:10.2147/LCTT.S192830.

15. Caccavari F, Valdembri D, Sandri C, Bussolino F, Serini G. Integrin signaling and lung cancer. Cell Adh Migr. 2010;4:124–9. doi:10.4161/cam.4.1.10976.

16. Marullo R, Ahn H, Cardenas M, Melnick A, Xue F, Cerchietti L. Abstract 1271: The transcription factor BCL6 is a rational target in non-small cell lung cancer (NSCLC). Cancer Res. 2016;76 14 Supplement:1271–1271. doi:10.1158/1538-7445.AM2016-1271.

17. Ding L, Wang H, Lang W, Xiao L. Protein kinase C-epsilon promotes survival of lung cancer cells by suppressing apoptosis through dysregulation of the mitochondrial caspase pathway. J Biol Chem. 2002;277:35305–13. doi:10.1074/jbc.M201460200.

18. Symonds JM, Ohm AM, Carter CJ, Heasley LE, Boyle TA, Franklin WA, et al. Protein kinase C δ is a downstream effector of oncogenic K-ras in lung tumors. Cancer Res. 2011;71:2087–97. doi:10.1158/0008-5472.CAN-10-1511.

19. Burkholder B, Huang R-Y, Burgess R, Luo S, Jones VS, Zhang W, et al.

Tumor-induced perturbations of cytokines and immune cell networks. Biochim Biophys Acta. 2014;1845:182–201. doi:10.1016/j.bbcan.2014.01.004.

20. Domagala-Kulawik J. The role of the immune system in non-small cell lung carcinoma and potential for therapeutic intervention. Transl Lung Cancer Res. 2015;4:177–90. doi:10.3978/j.issn.2218-6751.2015.01.11.

21. Chen W, Li Z, Bai L, Lin Y. NF-kappaB in lung cancer, a carcinogenesis mediator and a prevention and therapy target. Front Biosci (Landmark Ed). 2011;16:1172–85. doi:10.2741/3782.

22. Deng K, Liu L, Tan X, Zhang Z, Li J, Ou Y, et al. WIP1 promotes cancer stem cell properties by inhibiting p38 MAPK in NSCLC. Signal Transduct Target Ther. 2020;5:36. doi:10.1038/s41392-020-0126-x.

23. Bommhardt U, Schraven B, Simeoni L. Beyond TCR signaling: emerging functions of lck in cancer and immunotherapy. Int J Mol Sci. 2019;20. doi:10.3390/ijms20143500.

24. Peng C, Sun Q, Hao Y, Cong B, Zhao Y, Zhao X. Syk is low-expressed in non-small-cell lung cancer and inversely correlates with patient's survival. Acta Biochim Biophys Sin (Shanghai). 2013;45:149–51. doi:10.1093/abbs/gms102.

25. Chen J, Tan Y, Sun F, Hou L, Zhang C, Ge T, et al. Single-cell transcriptome and antigen-immunoglobin analysis reveals the diversity of B cells in non-small cell lung cancer. Genome Biol. 2020;21:152. doi:10.1186/s13059-020-02064-6.

26. Xia Y, Zhan C, Feng M, Leblanc M, Ke E, Yeddula N, et al. Targeting CREB pathway suppresses small cell lung cancer. Mol Cancer Res. 2018;16:825–32. doi:10.1158/1541-7786.MCR-17-0576.

27. Rodón L, Svensson RU, Wiater E, Chun MGH, Tsai W-W, Eichner LJ, et al. The CREB coactivator CRTC2 promotes oncogenesis in LKB1-mutant non-small cell lung cancer. Sci Adv. 2019;5:eaaw6455. doi:10.1126/sciadv.aaw6455.

28. Qian D, Liu H, Zhao L, Wang X, Luo S, Moorman PG, et al. Novel genetic variants in genes of the Fc gamma receptor-mediated phagocytosis pathway predict non-small cell lung cancer survival. Transl Lung Cancer Res. 2020;9:575–86. doi:10.21037/tlcr-19-318.

29. Li L, Wei Y, To C, Zhu C-Q, Tong J, Pham N-A, et al. Integrated omic analysis of lung cancer reveals metabolism proteome signatures with prognostic impact. Nat Commun. 2014;5:5469. doi:10.1038/ncomms6469.

30. Xu T, Li D, He Y, Zhang F, Qiao M, Chen Y. Prognostic value of metformin for non-small cell lung cancer patients with diabetes. World J Surg Oncol. 2018;16:60. doi:10.1186/s12957-018-1362-1.

31. Zhou R-H, Zhang J-T, Chen C, Xu Z-H, Lv X-B, Ye L, et al. Identification of CDC5L as bridge gene between chronic obstructive pulmonary disease and lung adenocarcinoma. Epigenomics. 2020. doi:10.2217/epi-2020-0112.

32. Siebring-van Olst E, Blijlevens M, de Menezes RX, van der Meulen-Muileman IH, Smit EF, van Beusechem VW. A genome-wide siRNA screen for regulators of tumor suppressor p53 activity in human non-small cell lung cancer cells identifies components of the RNA splicing machinery as targets for anticancer treatment. Mol Oncol. 2017;11:534–51. doi:10.1002/1878-0261.12052.

33. Ren J, Li X, Dong H, Suo L, Zhang J, Zhang L, et al. miR-210-3p regulates the proliferation and apoptosis of non-small cell lung cancer cells by targeting SIN3A. Exp Ther Med. 2019;18:2565–73. doi:10.3892/etm.2019.7867.

34. Suzuki H, Ouchida M, Yamamoto H, Yano M, Toyooka S, Aoe M, et al. Decreased expression of the SIN3A gene, a candidate tumor suppressor located at the prevalent allelic loss region 15q23 in non-small cell lung cancer. Lung Cancer. 2008;59:24–31. doi:10.1016/j.lungcan.2007.08.002.

35. Yan H, Bi L, Wang Y, Zhang X, Hou Z, Wang Q, et al. Integrative analysis of multi-omics data reveals distinct impacts of DDB1-CUL4 associated factors in human lung adenocarcinomas. Sci Rep. 2017;7:333. doi:10.1038/s41598-017-00512-1.

36. Yanagawa N, Osakabe M, Ogata SY, Shiono S. 43P MIB-1 labeling index is useful as prognostic and predictive markers for adjuvant therapy in non-small cell lung cancer. Ann Oncol. 2015;26:ix8. doi:10.1093/annonc/mdv518.18.

37. Zhang X, Zhang Y, Miao Y, Zhou H, Jiang G, Wang E. TMEM17 depresses invasion and metastasis in lung cancer cells via ERK signaling pathway. Oncotarget. 2017;8:70685–94. doi:10.18632/oncotarget.19977.

38. Kumar M, Matta A, Masui O, Srivastava G, Kaur J, Thakar A, et al. Nuclear heterogeneous nuclear ribonucleoprotein D is associated with poor prognosis and interactome analysis reveals its novel binding partners in oral cancer. J Transl Med. 2015;13:285. doi:10.1186/s12967-015-0637-3.

39. Brägelmann J, Böhm S, Guthrie MR, Mollaoglu G, Oliver TG, Sos ML. Family matters: How MYC family oncogenes impact small cell lung cancer. Cell Cycle. 2017;16:1489–98. doi:10.1080/15384101.2017.1339849.

40. Dang CV. c-Myc target genes involved in cell growth, apoptosis, and metabolism. Mol Cell Biol. 1999;19:1–11. doi:10.1128/mcb.19.1.1.

41. Love, Michael, Simon Anders, and Wolfgang Huber. "Differential analysis of count data–the DESeq2 package." Genome Biol 15.550 (2014): 10-1186.

42. Robinson, Mark D., Davis J. McCarthy, and Gordon K. Smyth. "edgeR: a Bioconductor package for differential expression analysis of digital gene expression data." Bioinformatics 26.1 (2010): 139-140.

43. Law, Charity W., et al. "voom: Precision weights unlock linear model analysis tools for RNA-seq read counts." Genome biology 15.2 (2014): R29.
